# Radial Wall Strain as an Angiography-Derived Biomarker for Plaque Vulnerability Assessment: Pathophysiology, Clinical Applications, and Prognostic Implications

**DOI:** 10.3390/jcm15145389

**Published:** 2026-07-09

**Authors:** Lisa Simioni, Wesley Bennar, Giulia S Beretta, Thaïs Pittet, Matteo Chal, Julius Jelisejevas, Giacomo Maria Cioffi, Pascal Meier, Mariama Akodad, Thomas Hovasse, Philippe Garot, Lorenzo Fargione, Nicolas Amabile, Peter Wenaweser, Mario Togni, Stéphane Cook, Ioannis Skalidis

**Affiliations:** 1 Department of Cardiology, HFR-Fribourg Cantonal Hospital and University, 1708 Fribourg, Switzerland; 2 Institut Cardiovasculaire Paris Sud, Hôpital Privé Jacques Cartier, Ramsay Santé, 91300 Massy, France

**Keywords:** radial wall strain, angiography-derived physiology, OCT, ACS, plaque vulnerability

## Abstract

Radial wall strain (RWS) is an angiography-derived biomechanical index that quantifies plaque deformability from routine coronary angiography, offering a novel approach to assess plaque vulnerability beyond conventional anatomical and physiological parameters. This narrative review synthesizes current evidence on the pathophysiological basis, clinical applications, and prognostic implications of RWS in coronary artery disease. Recent studies have demonstrated that elevated RWS values are associated with established markers of plaque vulnerability, including thin-cap fibroatheromas and large lipid cores, as well as increased risk of major adverse cardiovascular events. Furthermore, RWS has emerged as a promising tool for identifying vulnerable plaques in non-flow-limiting lesions and predicting in-stent restenosis, demonstrating incremental prognostic value when combined with angiography-derived physiological indices such as the quantitative flow ratio. By integrating biomechanical assessment with conventional physiological and morphological characterization, RWS may enhance risk stratification and guide clinical decision-making in percutaneous coronary intervention. While current evidence is encouraging, prospective randomized trials are needed to validate RWS-guided management strategies and establish optimal cutoff values for clinical implementation.

## 1. Introduction

Acute coronary syndrome (ACS) remains one of the leading causes of cardiovascular morbidity and mortality worldwide [1]. Since the introduction of selective coronary angiography by Mason Sones in 1958, this technique has become the gold standard for the anatomical assessment of coronary arteries [2,3]. It plays a central role in patient management by enabling both the diagnosis of coronary lesions and their immediate treatment through percutaneous coronary intervention (PCI).

However, despite its fundamental role, coronary angiography remains limited to a two-dimensional luminographic representation of the coronary vessels and does not allow precise characterization of atherosclerotic plaque composition [4,5]. In particular, it provides limited information regarding the biomechanical properties of the vascular wall or the mechanisms underlying plaque vulnerability, both of which are critical factors in the development of ACS [6,7].

Consequently, the angiographic severity of a coronary stenosis does not always correlate with its functional significance or its propensity for rupture. Advances in both histopathology and intracoronary imaging have enabled the identification of thin-cap fibroatheromas (TCFA), along with other high-risk plaque substrates—such as large lipid cores, macrophage infiltration, and microcalcifications—as major precursors of ACS [6,8,9]. These findings have highlighted the need to move beyond the simple anatomical assessment of the coronary lumen to better predict plaque vulnerability and the associated risk of adverse cardiovascular events. This has led to the development and widespread adoption of intracoronary imaging modalities capable of providing a detailed characterization of plaque morphology.

Intravascular ultrasound (IVUS) enables the assessment of plaque burden, vascular remodeling, and minimal lumen area, whereas optical coherence tomography (OCT) offers high-resolution visualization of plaque microstructure and fibrous cap thickness [10,11,12]. These imaging modalities now play an increasingly important role in optimizing percutaneous coronary interventions and in identifying vulnerable plaques [13,14,15]. However, both IVUS and OCT remain invasive techniques requiring dedicated intracoronary devices, additional procedural time, and increased costs, which limit their routine use in all patients undergoing coronary angiography. Furthermore, despite their ability to provide detailed morphological characterization of atherosclerotic plaques, no intracoronary imaging modality has yet been universally adopted as the reference standard for prospective plaque-guided management strategies.

In parallel, the field of coronary physiology has witnessed the emergence of angiography-derived indices such as the quantitative flow ratio (QFR), vessel fractional flow reserve (vFFR), angiography-derived fractional flow reserve (FFRangio), and the Murray law-based quantitative flow ratio (μQFR), enabling functional lesion assessment without the need for pressure wires or pharmacologically induced hyperemia [16,17,18,19]. These advances have highlighted the growing clinical interest in extracting both physiological and prognostic information directly from conventional coronary angiography. Nevertheless, while angiography-derived physiological indices have expanded the possibilities for non-invasive functional assessment, an unmet need remains for a similarly accessible approach capable of evaluating plaque vulnerability.

In this context, increasing attention has recently focused on the role of biomechanical forces acting on the arterial wall. Thus, angiography-derived Radial Wall Strain (RWS) has emerged as a promising marker of plaque vulnerability, as supported by several studies and meta-analyses [20,21,22]. RWS quantifies the cyclic variations in luminal diameter throughout the cardiac cycle along a coronary segment, thereby reflecting the mechanical properties and deformability of the vascular wall. Using a single angiographic projection, it is now possible to simultaneously calculate both RWS and the Murray law-based quantitative flow ratio (μQFR), enabling combined biomechanical and functional assessment during routine coronary catheterization. To the best of our knowledge, this is the first narrative review to specifically synthesize the growing body of evidence on angiography-derived RWS, providing an up-to-date overview of its pathophysiological basis, clinical applications, and prognostic implications for the interventional cardiology community.

## 2. Plaque Vulnerability and Future Risk of Acute Coronary Syndromes

Understanding the pathophysiological mechanisms underlying ACS largely relies on the study of vulnerable coronary atherosclerotic plaques. The development of atherosclerosis is a complex process involving endothelial dysfunction, chronic inflammation, lipid accumulation, and progressive vascular remodeling [23]. Although many atherosclerotic plaques remain clinically silent, some exhibit morphological and biological features that predispose them to rupture, erosion, or thrombosis.

The histopathological prototype of the vulnerable plaque is the thin-cap fibroatheroma (TCFA), as extensively described by Virmani et al. and others studies [24,25]. These plaques are characterized by a large lipid-rich necrotic core composed of cholesterol deposits, foam macrophages, and cellular debris, covered by a thin fibrous cap (<65 μm) [25]. They also exhibit significant macrophage infiltration, particularly at the plaque shoulders. Additional features include intraplaque neovascularization promoting intraplaque hemorrhage, spotty calcifications, and positive vascular remodeling, which preserves luminal dimensions despite a substantial atherosclerotic burden [25,26].

These lesions are currently considered the principal pathological substrate of ACS and are thought to account for approximately 60–70% of myocardial infarctions [27]. This observation has stimulated considerable interest in their early identification. Several intracoronary imaging studies have demonstrated an association between these vulnerability features and the risk of future cardiovascular events. In a post hoc analysis of the SCOT-HEART trial including 1769 patients with suspected stable coronary artery disease followed for a median of 4.7 years, the presence of at least one adverse plaque feature (positive remodeling or low-attenuation plaque) was associated with a threefold higher risk of coronary heart disease death or non-fatal myocardial infarction (4.1% vs. 1.4%; HR: 3.01, 95% CI: 1.61–5.63; *p* = 0.001) [28]. More recently, an updated meta-analysis of 12 adjusted observational studies including 8453 patients and 22,319 untreated coronary lesions confirmed that all major plaque vulnerability features independently predicted future MACE [29]. Among these markers, TCFA was associated with a threefold higher risk (HR: 3.06, 95% CI: 1.97–4.77) and minimal lumen area with an HR of 2.78 (95% CI: 1.84–4.20), while overall plaque burden emerged as the strongest predictor (HR: 3.92, 95% CI: 1.45–10.59) [29].

While vulnerable plaque characteristics have consistently been associated with future cardiovascular events, whether their identification should lead to prophylactic intervention remains debated. The randomized PREVENT trial enrolled 1606 patients with non-flow-limiting vulnerable plaques identified by intracoronary imaging, randomizing them to preventive PCI plus optimal medical therapy or optimal medical therapy alone [30]. At 2 years, preventive PCI significantly reduced the primary composite endpoint compared with optimal medical therapy alone (0.4% vs. 3.4%; absolute risk reduction 3.0%, 95% CI: 1.8–4.4; *p* = 0.0003). Nevertheless, longer-term follow-up is needed to confirm the durability of this benefit, and PREVENT represents an important milestone in the transition from vulnerable plaque identification toward targeted preventive treatment [30]. The clinical relevance of these findings has led to the development of intracoronary imaging techniques capable of accurately characterizing plaque morphology. IVUS enables the assessment of plaque burden, vascular remodeling, and minimal lumen area, whereas OCT provides detailed visualization of plaque microstructure owing to its superior spatial resolution [13]. These imaging modalities are now recommended by contemporary guidelines to guide selected complex coronary interventions and optimize stent implantation [31,32].

Atherosclerotic vulnerability, however, is not limited to native plaques. The development of in-stent neoatherosclerosis is now recognized as an important mechanism of restenosis and late stent thrombosis [33]. Histopathological studies from Virmani’s group demonstrated that these lesions share many characteristics with vulnerable native plaques, including lipid accumulation, persistent inflammation, and the presence of thin-cap fibroatheromas [34]. These observations have substantially advanced our understanding of the mechanisms underlying late stent failure.

Despite the importance of the vulnerable plaque concept, plaque vulnerability alone does not fully explain the occurrence of acute coronary events. As highlighted by Virmani and colleagues, the development of an acute event depends on the interaction between a vulnerable plaque, a prothrombotic milieu (“vulnerable blood”), and a myocardium susceptible to malignant arrhythmias (“vulnerable myocardium”) [27]. This integrative concept helps explain why some plaques exhibiting morphological features of vulnerability remain clinically silent for years, whereas others rapidly precipitate an acute coronary event.

These limitations have encouraged the development of novel approaches aimed at complementing conventional morphological plaque characterization. Among them, the assessment of biomechanical forces acting on the arterial wall appears particularly promising. By incorporating vessel deformation throughout the cardiac cycle, parameters such as RWS may help identify high-risk plaques beyond their anatomical composition alone and provide an additional dimension for coronary risk stratification.

## 3. Angiographic Biomechanics: Radial Wall Strain (RWS)

### 3.1. Pathophysiological Basis and Biomechanical Principles

**Figure 1:**
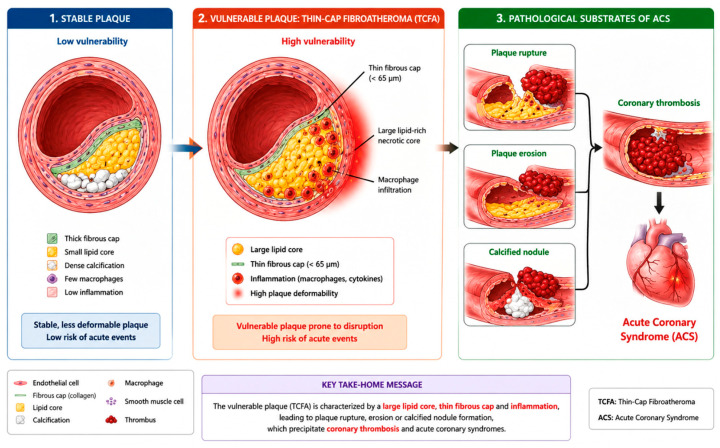
Histopathological determinants of plaque vulnerability and mechanisms leading to ACS. Landmark pathological studies identified TCFA, plaque rupture, plaque erosion, and calcified nodules as major substrates of coronary thrombosis. Beyond plaque composition, biomechanical properties contribute to plaque destabilization.

As described above, histopathological and intracoronary imaging studies have identified the TCFA and related features as the principal substrates of plaque vulnerability (Section 2) [6,8]. Beyond plaque composition and biological activity, these vulnerable substrates also exhibit distinct mechanical behavior, which forms the conceptual basis for biomechanical assessment of plaque stability (see Figure 1).

However, beyond plaque composition alone, biomechanical forces acting on the arterial wall also play a critical role in plaque destabilization and rupture. In this context, RWS has emerged as an innovative angiography-derived non-invasive biomarker capable of indirectly assessing the biomechanical properties of coronary plaques [21,35]. RWS quantifies the relative variation in luminal diameter throughout the cardiac cycle, reflecting the deformation capacity of the vascular wall in response to pulsatile forces generated by arterial pressure [22,35].

The pathophysiological basis of RWS relies on the premise that vulnerable plaques exhibit distinct mechanical properties compared with stable lesions. Lipid-rich plaques characterized by a thin fibrous cap are generally more deformable, whereas fibrotic and heavily calcified plaques tend to be mechanically stiffer and therefore less susceptible to cyclic dimensional changes during the cardiac cycle [35,36]. Consequently, elevated RWS values reflect increased local compliance and may serve as an indirect marker of plaque vulnerability.

Unlike physiological indices such as FFR and iFR, which are invasive pressure-derived measurements primarily designed to assess the hemodynamic significance of a coronary stenosis, RWS explores the biomechanical dimension of coronary artery disease [22]. As such, it provides complementary information regarding plaque instability and rupture risk, independent of the ischemic burden imposed by the lesion [35].

### 3.2. Angiography-Derived Radial Wall Strain: Definition and Calculation

Radial Wall Strain (RWS) is an angiography-derived biomechanical index that quantifies coronary wall deformation induced by pulsatile pressure changes throughout the cardiac cycle. This deformation is directly dependent on tissue stiffness and, therefore, on the underlying plaque composition. Vulnerable plaques, characterized by a high lipid content and significant inflammatory infiltration, generally exhibit higher RWS values than stable fibrotic or calcified plaques. Consequently, RWS represents an indirect marker of plaque vulnerability that can be obtained from conventional coronary angiography without the need for additional intracoronary instrumentation.

This parameter is entirely derived from conventional coronary angiography and does not require pressure wires, hyperemic agents, or additional intracoronary imaging catheters. Nevertheless, reliable analysis requires high-quality angiographic acquisitions obtained at a frame rate of at least 15 frames per second, with minimal vessel foreshortening and overlap [35,36]. As a two-dimensional technique, RWS cannot capture the three-dimensional, circumferential distribution of plaque deformation that can be assessed by IVUS- or OCT-based strain analysis. It also does not characterize plaque composition directly, and its accuracy remains dependent on angiographic image quality, projection angle, and the absence of vessel foreshortening or overlap.

Because RWS is calculated from the overall change in luminal diameter at a given cross-section, it does not differentiate between circumferential and eccentric plaque morphology. In eccentric plaques, only a portion of the vessel wall is occupied by atherosclerotic tissue, while the remaining wall segment retains normal compliance. As a result, the measured RWS may reflect a composite of plaque and residual normal-wall deformability, potentially underestimating the true biomechanical stiffness of the diseased segment. The minimum proportion of circumferential wall involvement required to meaningfully influence the RWS signal has not been established, and this represents an important area for future methodological research, ideally through co-registration with cross-sectional imaging (IVUS/OCT) capable of quantifying plaque arc.

**Figure 2:**
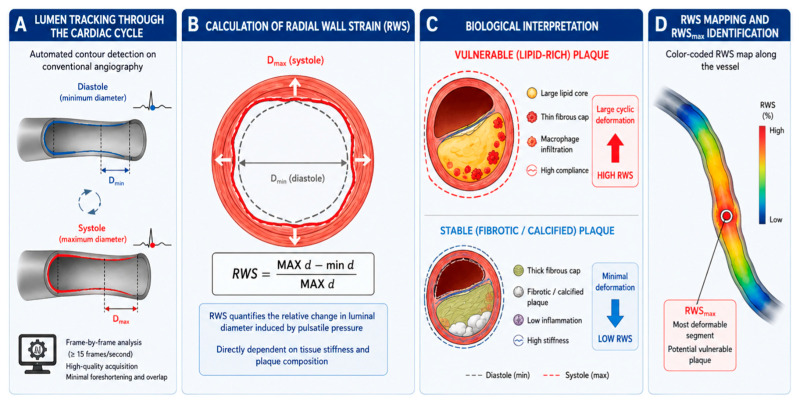
Angiography-Derived RWS: Acquisition and Biological Significance. Illustration of the workflow for RWS analysis, shifting from automated lumen contour tracking throughout the cardiac cycle to the calculation of local vessel deformation and the identification of the maximal strain region (RWSmax). Higher RWS values are associated with lipid-rich, compliant plaques, whereas lower values characterize fibrotic or calcified lesions, supporting the role of RWS as an angiography-derived marker of plaque vulnerability.

Angiography derived RWS analysis relies on automated software algorithms that reconstruct luminal vessel contours throughout different phases of the cardiac cycle. Following the identification of systolic and diastolic frames, local variations in luminal diameter are quantified along the entire coronary segment. The highest strain value measured within the analyzed vessel is defined as the maximal radial wall strain (RWSmax), which is typically located at the site of the stenosis or within the most deformable plaque region (see Figure 2).

### 3.3. Relationship with Plaque Vulnerability and Intracoronary Imaging Findings

To evaluate the potential clinical utility of this novel biomarker, several studies have investigated the ability of radial RWS to identify vulnerable plaques by comparing it with established intracoronary imaging modalities, particularly OCT and intravascular ultrasound combined with near-infrared spectroscopy (IVUS-NIRS) [20,36]. Overall, these studies have consistently demonstrated a significant association between elevated RWS values and morphological features recognized as markers of plaque vulnerability.

OCT-based investigations have shown a positive correlation between RWS and plaque lipid burden, the lipid-to-cap ratio, and the presence of TCFAs [21]. Conversely, RWS has been found to correlate negatively with fibrous cap thickness, suggesting that the most deformable lesions are also those exhibiting the highest-risk morphological characteristics. These findings support the hypothesis that increased plaque deformability reflects underlying structural instability and a greater propensity for future plaque disruption [21].

More recently, studies incorporating IVUS-NIRS have further validated these observations by demonstrating a strong ability of RWS to identify high-risk plaque phenotypes [37,38]. Interestingly, the discriminatory performance of RWS for the detection of vulnerable plaques appears to exceed that of purely physiological indices such as μQFR, emphasizing the complementary nature of biomechanical and functional assessments [35,36].

Collectively, these findings suggest that RWS does not merely reflect the anatomical severity or physiological significance of a coronary stenosis but also captures biomechanical information directly related to plaque structural integrity and vulnerability. As such, RWS provides a unique dimension of lesion assessment that is not accessible through conventional physiological measurements alone.

Although RWS is a relatively recent angiography-derived parameter, emerging evidence suggests that its diagnostic performance for identifying vulnerable plaques may be comparable to those of established intracoronary imaging modalities such as IVUS and OCT [21,37,39]. More importantly, rather than replacing these techniques, RWS appears to provide complementary biomechanical information that cannot be directly assessed by current imaging or physiological approaches [21]. Consequently, the integration of RWS with intracoronary imaging and physiological assessment may enable a more comprehensive characterization of coronary lesions and improve the identification of plaques at increased risk of future adverse cardiovascular events.

### 3.4. Prognostic Value and Clinical Applications in PCI

Beyond its diagnostic utility, RWS has demonstrated significant prognostic value across several populations of patients with coronary artery disease. Follow-up studies have consistently shown that elevated RWS values are independently associated with an increased risk of myocardial infarction, MACE, and target vessel-related adverse outcomes. In a matched case–control study of mild-to-intermediate lesions, a baseline RWSmax > 12% was present in 77.3% of lesions that subsequently caused acute myocardial infarction versus 15.2% of matched controls, corresponding to an adjusted risk ratio of 7.25 (95% CI: 3.94–13.37; *p* < 0.001) [22]. In the FAVOR III China cohort of non-flow-limiting vessels with preserved physiology, the 1-year rate of the vessel-oriented composite endpoint was 14.3% in vessels with RWSmax > 12% versus 2.9% in those with RWSmax ≤ 12% (adjusted HR: 4.44, 95% CI: 2.43–8.14; *p* < 0.001) [35]. Similarly, among diabetic patients with non–flow-limiting stenosis from the COMBINE OCT-FFR trial, RWS-positive patients (RWSmax ≥ 13.0%) had a 5-year lesion-oriented composite event rate of 17.0% versus 6.8% in RWS-negative patients (HR: 2.70, 95% CI: 1.37–5.32; *p* = 0.004), with RWS remaining an independent predictor after multivariable adjustment (adjusted HR: 2.88, 95% CI: 1.35–6.15; *p* = 0.006) [39]. These findings highlight the independent and reproducible prognostic information provided by this novel biomarker across distinct clinical populations.

One of the most clinically relevant observations concerns non-ischemic coronary lesions. Among vessels with preserved coronary physiology, patients exhibiting high RWS values experience substantially higher rates of adverse cardiovascular events compared with those presenting low RWS values [35]. Conversely, the combination of a normal μQFR and a low RWS identifies a subgroup of patients at particularly low risk of future cardiovascular events, further supporting the safety of deferring revascularization in appropriately selected lesions [35,36,39].

Several studies have also demonstrated that RWS provides prognostic information that is independent of FFR and of plaque vulnerability features identified by intracoronary imaging modalities such as IVUS and OCT [36,39]. This independence suggests that RWS captures a specific biomechanical component of atherosclerotic disease that is not directly assessed by conventional physiological or morphological techniques. Consequently, RWS may offer incremental value when integrated into contemporary lesion assessment strategies.

Moreover, methodological studies have demonstrated excellent reproducibility of RWS measurements. Intra- and interobserver intraclass correlation coefficients of 0.92 (95% CI: 0.84–0.96) and 0.90 (95% CI: 0.81–0.95) have been reported in one validation cohort [22], with similarly high agreement rates of 0.90 (95% CI: 0.80–0.95) and 0.87 (95% CI: 0.75–0.94) in an independent diabetic cohort [39]. Analysis time was consistently under one minute per lesion [21,22,35,36]. These characteristics support its potential integration into routine clinical practice without significantly prolonging catheterization procedures. Furthermore, RWS can be calculated simultaneously with μQFR from a single angiographic acquisition, allowing combined biomechanical and functional assessment of coronary lesions. This multiparametric approach represents one of the most promising developments in contemporary angiographic analysis, offering a more comprehensive evaluation of coronary artery disease by integrating both plaque vulnerability and physiological significance within the same diagnostic framework.

### 3.5. Clinical Applications of RWS in Stent Failure and Drug-Coated Balloon Therapy

Emerging evidence further suggests a potential role for RWS in predicting in-stent restenosis following percutaneous coronary intervention [40]. Lesions characterized by elevated pre-procedural RWS values appear to be associated with a higher risk of subsequent restenosis, possibly reflecting the complex interplay between local inflammation, vascular remodeling, and persistent biomechanical stress despite successful stent implantation [40,41].

Beyond its potential role in the identification of vulnerable plaques, RWS has also attracted increasing interest as a prognostic stratification tool and a potential aid in clinical decision-making during PCI. Indeed, the biomechanical properties of atherosclerotic plaques influence not only their susceptibility to rupture but also their response to different therapeutic strategies. This concept has led to the investigation of RWS across various interventional settings, including stent-less approaches based on the use of drug-coated balloons (DCBs).

In this context, Genta et al. evaluated the prognostic value of RWS in 264 de novo coronary lesions treated exclusively with DCBs [42]. The study demonstrated a significant reduction in RWS following lesion preparation and DCB treatment, suggesting that angioplasty alters the biomechanical properties of both the plaque and the vessel wall. This observation is consistent with the mechanical remodeling induced by vessel dilation and supports the concept that RWS may serve as a dynamic marker of structural changes occurring during PCI. The authors also assessed the predictive performance of RWS at different stages of the procedure. Among the various measurements analyzed, post-predilation RWS showed the highest discriminative ability for predicting MACE [42].

However, unlike μQFR, which emerged as an independent predictor of MACE after adjustment for confounding factors, RWS did not demonstrate significant prognostic value in this cohort. No statistically significant difference in event-free survival was observed between patients with high and low RWS values. These findings suggest that, in lesions treated with DCBs, functional parameters directly assessing the hemodynamic consequences of revascularization may be more relevant for predicting clinical outcomes than biomechanical markers reflecting plaque composition. This divergence may reflect two non-mutually exclusive mechanisms: controlled plaque modification during DCB angioplasty (vessel dilation and micro-dissection) may disrupt the local elasticity vectors that RWS depends on, rendering post-procedural strain measurements less representative of residual plaque biology; alternatively, once the hemodynamic obstruction has been corrected, the physiological index μQFR may simply become the dominant determinant of short-term outcomes, irrespective of any change in biomechanical wall properties. Regardless of the underlying mechanism, this finding suggests that the clinical utility of RWS may be primarily confined to pre-procedural plaque characterization rather than post-procedural outcome prediction. These findings suggest that RWS may complement plaque assessment and contribute to a better understanding of lesion response to interventional therapies. Nevertheless, its prognostic value still requires confirmation in larger prospective multicenter studies. These findings suggest that RWS utility may be primarily pre-procedural for plaque vulnerability assessment rather than post-intervention for outcome prediction.

### 3.6. Combined RWS and μQFR Assessment for Coronary Risk Stratification

The integration of biomechanical and physiological plaque assessment has emerged as a promising strategy for improving coronary risk stratification. In this context, Chu et al. investigated the relationship between angiography-derived RWS and serial changes in coronary physiology assessed by QFR in 175 medically treated intermediate coronary lesions [43]. The authors hypothesized that plaques exhibiting greater biomechanical instability would be more likely to undergo functional deterioration over time. RWSmax was measured on baseline angiograms, while lesion-specific QFR changes were assessed during a median follow-up of 12.4 months. Lesions with subsequent physiological progression demonstrated significantly higher baseline RWSmax values compared with stable lesions (11.8% vs. 10.8%; *p* = 0.001), and a high RWSmax (>12%) was more frequent among progressing lesions (47.6% vs. 20.5%; *p* < 0.001). After adjustment for clinical and angiographic risk factors, a high RWSmax remained an independent predictor of functional lesion progression (adjusted OR: 2.871, 95% CI: 1.343–6.138; *p* = 0.007). Notably, RWSmax outperformed conventional angiographic stenosis severity in predicting physiological deterioration (AUC: 0.658 vs. 0.533; *p* = 0.021). These findings support the concept that biomechanical plaque vulnerability contributes to the progression of coronary dysfunction before the occurrence of overt clinical events. By integrating information on both plaque biology and lesion-specific ischemic burden, this approach may improve the identification of lesions at risk of future progression and adverse cardiovascular outcomes.

Taken together, these findings support the concept that RWS is an innovative angiography-derived biomarker capable of integrating the biomechanical dimension of coronary artery disease. Complementing anatomical, physiological, and intracoronary imaging assessments, RWS has the potential to contribute to a more comprehensive and personalized evaluation of coronary lesions. Although current evidence is highly encouraging, prospective multicenter studies and randomized clinical trials are still required to determine whether RWS-guided therapeutic strategies can translate into improved long-term clinical outcomes.

Importantly, the growing body of evidence suggests that RWS should not be viewed as a replacement for established intracoronary imaging modalities such as IVUS and OCT, but rather as a complementary tool providing unique biomechanical information that is not captured by current anatomical or physiological assessments. The integration of RWS with coronary physiology and intracoronary imaging may therefore represent a promising multimodal approach for improving risk stratification, identifying vulnerable plaques, and optimizing clinical decision-making in contemporary interventional cardiology.

## 4. Discussion

Over the past two decades, the management of coronary lesions has evolved significantly. This progress is primarily due to advances in coronary physiology and intracoronary imaging techniques. Indeed, although conventional angiography remains the cornerstone for the anatomical assessment of coronary artery disease, this technique has limitations in assessing the individual risk associated with each atherosclerotic plaque. These limitations have therefore led to the development of new techniques that allow for a more comprehensive functional and biological characterization of coronary artery disease.

Development of physiological indices primarily assess stenosis’s ability to induce myocardial ischemia, rather than its propensity for destabilization. However, ischemia and plaque vulnerability do not necessarily represent the same dimensions of coronary risk. Several studies have demonstrated that a substantial proportion of ACS events arise from lesions that are minimally obstructive or non-ischemic but exhibit morphological characteristics that promote plaque rupture or erosion [44,45]. This dissociation between functional severity and the risk of adverse clinical events has driven the development of imaging techniques aimed at characterizing arterial wall structure and plaque vulnerability. Among these modalities are IVUS, OCT and, more recently, biomechanical approaches such as the assessment of RWS.

IVUS and OCT have significantly improved our understanding of coronary artery disease. IVUS provides excellent visualization of vascular architecture and arterial remodeling, while OCT allows for detailed assessment of plaque microstructure thanks to its exceptional spatial resolution [36,39]. These invasive imaging modalities have demonstrated considerable value in optimizing PCI and identifying high-risk plaques. Nevertheless, their use remains invasive, requires additional equipment, and is limited to selected clinical settings. Furthermore, while they accurately characterize plaque composition, they do not directly assess the biomechanical forces acting upon the plaque.

In this context, the emergence of RWS represents a particularly promising development. Unlike physiological indices, which assess the functional impact of stenosis, and imaging modalities, which describe plaque morphology, RWS explores the biomechanical dimension of coronary artery disease. This approach is based on the concept that plaque vulnerability depends not only on its composition but also on its response to the mechanical stresses generated across the cardiac cycle. Lipid-rich plaques with TCFA appear more deformable and are therefore more likely to exhibit elevated RWS values.

The currently available evidence supports this hypothesis. Hong et al. [21] demonstrated a significant association between RWS and several established markers of plaque vulnerability, including lipid burden, thin-cap fibroatheroma, and fibrous cap thinning as assessed by OCT. These results suggest that RWS may serve as an indirect angiographic marker of plaque structural instability. Notably, this information is obtained from conventional coronary angiography without the need for additional intracoronary devices.

Beyond its association with plaque morphology, several studies have also highlighted the prognostic value of RWS. Tu et al. [35] and Li et al. [22] reported that elevated RWS values were associated with an increased risk of MACE and myocardial infarction, even in patients with functionally non-significant lesions. These observations are particularly relevant because they suggest that biomechanical vulnerability may represent an independent risk factor for myocardial ischemia. Thus, two lesions with similar physiological profiles could have different prognoses depending on their biomechanical behavior. This complementarity between physiology, imaging, and biomechanics is also illustrated by the study conducted by Yang et al. [36], which demonstrated that RWS was associated with both the functional significance of coronary lesions and morphological characteristics of plaque vulnerability. These results reinforce the notion that RWS is not merely a substitute for existing physiological indices or imaging modalities but rather provides additional information that may improve risk stratification. Furthermore, the studies cited above also assessed inter- and intra-observer variability and reported low variability with satisfactory reproducibility.

More recently, the scope of RWS has expanded into the field of interventional cardiology. Studies by Zhang et al. [41] and Skalidis et al. [46] identified an association between elevated RWS values and the risk of in-stent restenosis. These findings suggest that local biomechanical forces may continue to influence vascular remodeling even after apparently optimal revascularization. This observation opens new perspectives for the early identification of patients at risk of percutaneous treatment failure following coronary stent implantation. Thus, measuring RWS in treated vessels may contribute to the development of more individualized therapeutic strategies and facilitate the identification of patients with persistent high-risk coronary profiles, even in secondary prevention.

The overall body of evidence is further strengthened by the recent meta-analysis conducted by Skalidis et al. [20], which confirmed the association between elevated RWS values and adverse cardiovascular outcomes. Although the number of available studies remains limited, the consistency of findings across different populations and clinical settings supports the robustness of the prognostic signal associated with high RWS values.

## 5. Challenges

Several challenges should be acknowledged. First, most of the currently available evidence is derived from observational studies conducted in relatively selected, high-risk patient populations, which may limit the generalizability of the findings. In addition, because RWS calculation depends on the diameter change driven by pulsatile pressure across the cardiac cycle, transient hemodynamic disturbances at the time of angiography (such as severe hypertension or hypotension, marked bradycardia or tachycardia, and arrhythmias) could theoretically affect measurement reproducibility, although this has not yet been systematically studied. Furthermore, the cited validation studies did not mandate the routine administration of intracoronary nitrates prior to RWS acquisition, and whether vasodilator pretreatment is required for standardized measurement remains to be clarified in future methodological work. To date, RWS has only been validated and applied clinically using a single integrated angiographic platform capable of computing RWS and μQFR simultaneously from the same projection. Whether other platforms will incorporate comparable strain modules, and whether RWS values remain consistent across implementations, remains to be established.

Second, optimal RWS thresholds have not yet been fully standardized, and the reproducibility of measurements may be influenced by the software platform used, as well as by angiographic image quality, given the technique’s reliance on two-dimensional angiographic reconstruction. In addition, although RWS has consistently demonstrated prognostic value across several studies, its incremental benefit over established physiological and imaging markers remains to be fully elucidated. Finally, no prospective randomized trial has yet demonstrated that an RWS-guided management strategy translates into improved clinical outcomes compared with contemporary standard-of-care approaches.

Despite these limitations, RWS represents one of the most promising advances in the field of angiography-derived plaque assessment. By providing a biomechanical perspective that is not captured by conventional angiography and only partially addressed by current physiological indices, RWS may offer complementary information regarding plaque vulnerability and future cardiovascular risk. As evidence continues to accumulate, the integration of biomechanical assessment with coronary physiology and intracoronary imaging may contribute to a more comprehensive characterization of coronary artery disease. Ultimately, a multimodal strategy combining physiological, morphological, and biomechanical parameters could enable more refined risk stratification and support increasingly personalized revascularization and preventive treatment strategies.

## 6. Conclusions

Successive advances in coronary physiology and intracoronary imaging have significantly improved the selection of patients eligible for revascularization and our understanding of the mechanisms underlying plaque vulnerability. Nevertheless, the persistence of cardiovascular events in patients with non-ischemic or morphologically mild lesions underscores the need for new risk stratification tools. In this context, RWS emerges as an innovative angiographic biomarker that incorporates a biomechanical dimension previously under-explored in clinical practice. Currently available data show consistent associations between high RWS, vulnerable plaque characteristics, the occurrence of major coronary events, and the risk of in-stent restenosis. Although the evidence remains limited and requires large-scale prospective validation, RWS could serve as a relevant complement to physiological indices and intracoronary imaging modalities. Its integration into a multimodal strategy combining anatomy, physiology, and biomechanical stress represents a promising approach.

## Data Availability

No new data were generated in this manuscript.

## Abbreviations

The following abbreviations are used in this manuscript:

RWS	Radial Wall Strain
ACS	Acute coronary Syndrome
PCI	Percutaneous coronary intervention
STEMI	ST-elevation myocardial infarction
NSTEMI	Non-ST-elevation myocardial infarction
OCT	Optical Coherence Tomography
IVUS	Intravascular Ultrasound
FFR	Fractional Flow Reserve
iFR	Instantaneous Wave-Free Ratio
RFR	Resting Full-Cycle Ratio
TCFA	Thin-cap fibroatheroma
MACE	Major adverse cardiovascular events
